# A Status Review of the Bioactive Activities of Tiger Milk Mushroom *Lignosus rhinocerotis* (Cooke) Ryvarden

**DOI:** 10.3389/fphar.2017.00998

**Published:** 2018-01-15

**Authors:** Neeranjini Nallathamby, Chia-Wei Phan, Syntyche Ling-Sing Seow, Asweni Baskaran, Hariprasath Lakshmanan, Sri N. Abd Malek, Vikineswary Sabaratnam

**Affiliations:** ^1^Mushroom Research Centre, University of Malaya, Kuala Lumpur, Malaysia; ^2^Department of Pharmacy, Faculty of Medicine, University of Malaya, Kuala Lumpur, Malaysia; ^3^Department of Biochemistry, Karpagam Academy of Higher Education, Coimbatore, India; ^4^Faculty of Science, Institute of Biological Sciences, University of Malaya, Kuala Lumpur, Malaysia

**Keywords:** *Lignosus rhinocerotis*, medicinal mushroom, sclerotium, medicinal properties, neuroprotection, antioxidant, ethnomedicine, mycomedicine

## Abstract

Edible and medicinal mushrooms are regularly used in natural medicines and home remedies since antiquity for ailments like fever, inflammation, and respiratory disorders. *Lignosus rhinocerotis* (Cooke) Ryvarden is a polypore found in Malaysia and other regions in South East Asia. It can be located on a spot where a tigress drips milk while feeding, hence the name “tiger's milk mushroom.” The sclerotium of *L. rhinocerotis* is highly sought after by the native communities in Malaysia to stave off hunger, relieve cough and asthma, and provide stamina. The genomic features of *L. rhinocerotis* have been described. The pharmacological and toxicity effects, if any, of *L. rhinocerotis* sclerotium have been scientifically verified in recent years. In this review, the validated investigations including the cognitive function, neuroprotection, immune modulation, anti-asthmatic, anti-coagulation, anti-inflammatory, anti-microbial/ anti-viral, anti-obesity, anti-cancer/ anti-tumor, and antioxidant properties are highlighted. These findings suggest that *L. rhinocerotis* can be considered as an alternative and natural medicine in the management of non-communicable diseases. However, there is a paucity of validation studies including human clinical trials of the mycochemicals of *L. rhinocerotis*.

## Introduction

**Figure 1:**
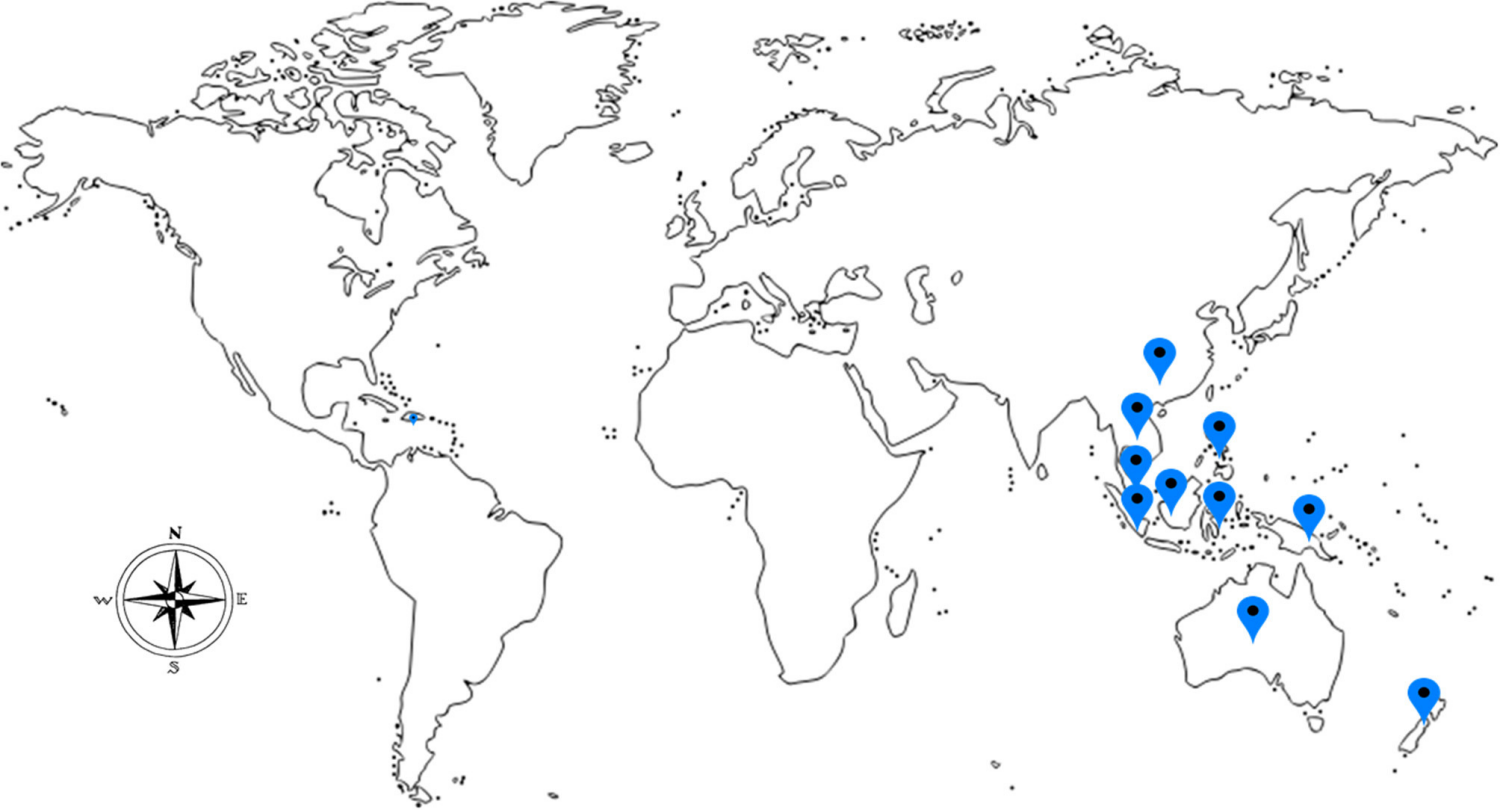
The geographical locations for the occurrence of *L. rhinocerotis*.

Medicinal mushrooms have been valued and used since ancient times by the Chinese, Korean, Japanese, Egyptians, and European communities. They are valued not only for the culinary purposes but also for their nutritional and medicinal values (Manzi et al., 1999). The greatest attribute of mushrooms, besides their taste, is their peculiar healing properties. Recently, ethnomycological knowledge of medicinal mushrooms for their curative properties is being tapped. *Lignosus rhinocerotis* (Cooke) Ryvarden, belonging to the Polyporaceae family is regarded as a rare and valuable traditional medicine and it can only be located in a certain geographic regions encompassing South China, Thailand, Malaysia, Indonesia, Philippines, Papua New Guinea, New Zealand, and Australia (Lai et al., 2011; Figure 1).

*Lignosus rhinocerotis*, however, was only collected from the wild. In the wild, the tiger milk mushroom grows solitary and makes the collection process time and energy consuming (Abdullah et al., 2013). Since the 2000s, large-scale cultivation of *L. rhinocerotis* in a controlled environment was made successful in Malaysia, overcoming the cost and supply problem (Lau et al., 2011, 2013a, 2015). Commercialization of this mushroom was then made possible and this opened the opportunity to investigate the potential pharmacological and nutraceutical properties of this mushroom for functional food or dietary supplements.

## Description of *L. rhinocerotis*

**Figure 2:**
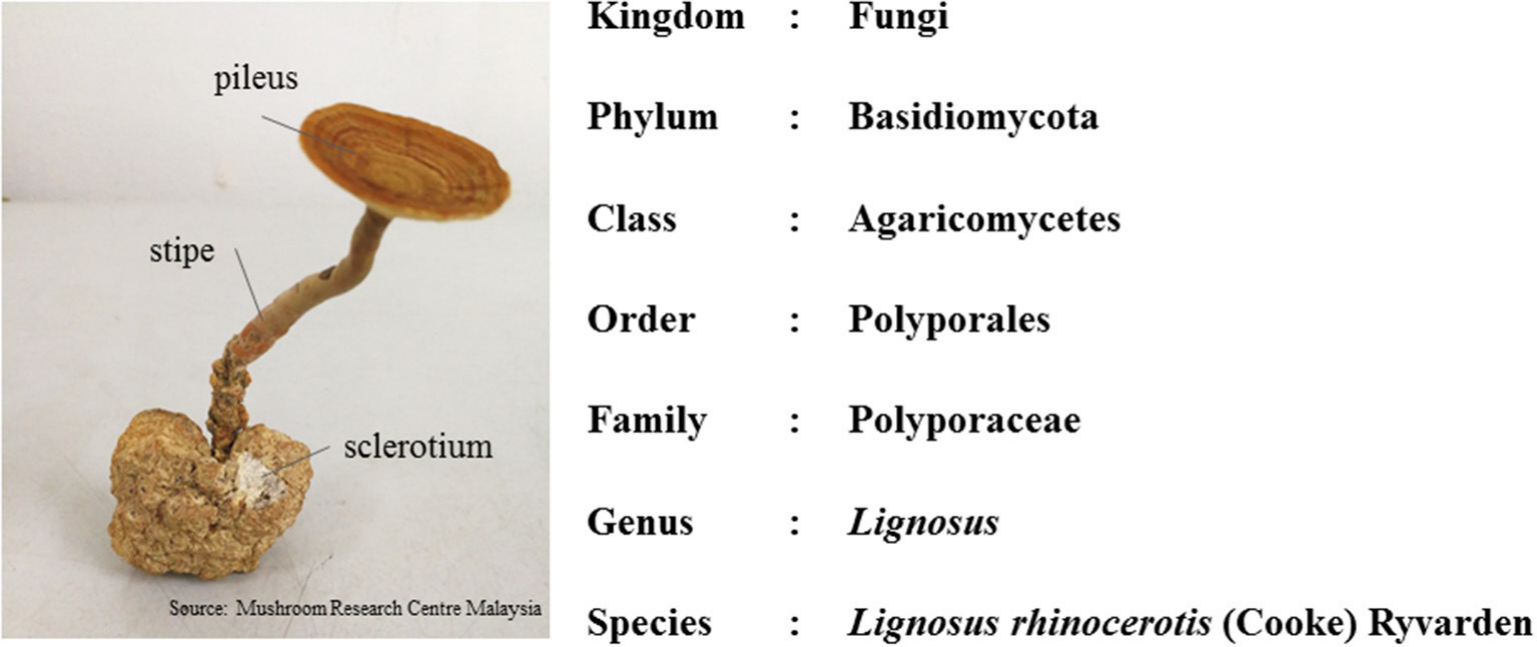
The morphology of *L. rhinocerotis* and its taxonomic classification.

This mushroom consists of the pileus (cap), stipe (stem), and sclerotium (tuber) (Figure 2). Its morphology is unusual for a polypore as the fruiting body (cap and stem) raises from the tuber under the ground, rather than from woody substrate. The cap and stem are woody while the sclerotium is a compacted mass of fungal mycelium containing food reserves. The sclerotium is white and gives a milk-like solution; and it even tastes like milk (Tan et al., 2010). As the irregular shaped sclerotium remains underground, the collection of the mushroom is challenging. Thus, due to lack of samples, limited study is done on this national treasure.

In Malaysia and Indonesia, this mushroom is known as “cendawan susu rimau” which literally means “tiger milk mushroom” (Burkill, 1930). Early documentation of this mushroom were given by Ridley and Corner (Ridley, 1900; Corner, 1989). In the early twentieth century, the taxonomy of *L. rhinocerotis* depended primarily on morphological observations. However, in recent years, specimens of “*susu rimau”* collected in these regions were confirmed as *L. rhinocerotis* on the basis of both micro- and macro-morphological characteristics, as well as molecular approaches. To date, *L. rhinocerotis* is noted to be the “most commonly occurring member of *Lignosus* in Malaysia” (Lai et al., 2009; Tan et al., 2010; Choong et al., 2014). Figure 2 shows the taxonomic classification of *L. rhinocerotis*.

*Lignosus rhinocerotis* was originally categorized as *Polyporus rhinoceros* (Cooke, 1879). This mushroom was taxonomically narrated under several genera, including *Fomes, Scindalma, Polystictus*, and *Microporus* by different authors before they were correctly re-named as *Lignosus* (Cooke, 1879). Some of the synonyms are “*Polyporus rhinocerus* Cooke (1879), *Fomes rhinocerotis* Cooke (1879), *Fomes rhinocerus* Cooke (1879), *Scindalma rhinocerus* (Cooke) Kuntze (1898), *Scindalma rhinocerotis* (Cooke) Kuntze (1898), *Polyporus sacer var. rhinocerotis* (Cooke) Llyod (1921), *Polystictus rhinocerus* (Cooke) Boedjin (1940), *Polystictus rhinocerotis* (Cooke) Boedjin (1940), *Microporus rhinocerus* (Cooke) Imazeki (1952), *Microporus rhinocerotis* (Cooke) Imazeki (1952), and *Lignosus rhinocerus* (Cooke) Ryvarden (1972)”. These synonyms were taken from MycoBank (http://www.mycobank.org).

*Lignosus rhinocerotis* was often mistaken for *Pleurotus tuber-regium* or *Lentinus tuber-regium*. Thus, the use of molecular markers in identifying *L. rhinocerotis* has proven to overcome the drawback of classic taxonomy methods (Sotome et al., 2008; Cui et al., 2011). There are even genetic markers and DNA barcode markers being developed to identify the *Lignosus* spp.

Recently, Tan et al. (2015) reported successful cultivation of the mushroom, which would overcome the supply problem and make possibilities for more investigation to be done on *L. rhinocerotis*. Other researchers also cultivated this mushroom using mycelium in submerged culture techniques. Further, the optimization of substrate formulation was done for the cultivation of *L. rhinocerotis* in conditions that mimic their natural environment. Based on the optimized formulation, the pilot cultivation was conducted. Both sclerotia and sporophores were successfully produced (Abdullah et al., 2013).

### Ethnomycological aspects of *L. rhinocerotis*

According to folklore, it is believed that the mushroom emerges on the spot where the milk of a tigress had accidentally dribbled during lactation. The sclerotium of the mushroom resembles the “congealed white mass of milk” (Corner, 1989; Chang, 2015). Different tribal communities in Malaysia have different referral names, such as “*betes kismas”* by the Semai (Chang and Lee, 2010), “*tish am ong”* by the Kensiu (Mohammad et al., 2012), and “*Pěti' Aa”* by the Besisi (Skeat, 1896). The Batak people from Indonesia denoted *L. rhinocerotis* as “*Ndurabi”* (Karo) (Hilton and Dhitaphichit, 1978). *Lignosus rhinocerotis* found in China were called “*how gui kou* or *hurulingzhi”* (in Chinese), which means “tiger milk *Ganoderma*” (Huang, 1999; Yokota, 2011). In Japan, it is known as “*hijiritake”* (Lee and Chang, 2007).

Besides the traditional beliefs that *L. rhinocerotis* was derived from the tiger's milk, there are many other folklore beliefs about this mushroom. The Semai (indigenous people of Malaysia) believes that *L. rhinocerotis* could reinstate the spirit of a crop and guarantee a lavish harvest. The sclerotia are habitually used during paddy farming and prayer ritual for a bountiful crop yields (Haji Taha, 2006). Alternatively, some crops, for instance, paddy is positioned in a flower-filled container, and suspended over the mushroom (Skeat and Ottoblagoen, 1906).

The Besisi (or MahMeri) declares that the mushroom is easier to be spotted after the full moon (Hartland, 1909). *Lignosus rhinocerotis* were also associated with childbirth customs. The Semang (or Negrito) people believe that *L. rhinocerotis* hold “the soul of an unborn tiger cub” and that “the soul is conveyed when the tiger eats it” (Cumming, 1903; Robert, 1986).

*Lignosus rhinocerotis* is mentioned in various stories from different cultural background, and in most cases, are linked to their medicinal properties. For example, in the story of “Indra Bangsawan”, “tigress' milk” is the remedy for a princess who became infected with an eye disease and lost her vision (Beveridge, 1909). A Mughal emperor, Jahangir (1568–1616) also wrote that the “milk of a tigress was of great use for brightening eyes” (Green, 2006; Lee et al., 2009).

Based on ethnobotanical uses, various tribes of the Orang Asli in Malaysia (the Semai, Temuan, and Jakun) use *L. rhinocerotis* to relief asthma, cough, food poisoning, swollen breasts, joint pain, liver illness, swollen body parts, and as a general tonic (Lee and Chang, 2007; Ismail, 2010). It is also used to nurse women after childbirth (during postpartum period) and also to stave off hunger. The *L. rhinocerotis* is not only popular among the indigenous people but also the urban population in Malaysia (Hattori et al., 2007). The sclerotia of *L. rhinocerotis* are occasionally sold in Traditional Chinese Medicine (TCM) store in Malaysia. They are used by the TCM practitioner to revitalize the body of the patients. According to Sabaratnam et al. (2013), infusions of *L. rhinocerotis* are said to improve the overall wellness of the individual by enhancing the vitality, energy, and alertness.

There are many ways this mushroom is prepared and consumed to treat illness. In earlier years, the sclerotium was pounded and the juice was infused with water and drunk as a tonic. The mushrooms are grounded or sliced, then boiled with water for drinking or soaked into Chinese wine for external applications (Chang and Lee, 2004). The sclerotium is also eaten raw and with betel leaves to relieve a cough and sore throat. The preparation methods of decoction and/or topical medicine vary among tribes.

### Genomic and proteomic studies of *L. rhinocerotis*

Genome sequencing of *L. rhinocerotis* was carried out (Yap et al., 2014). A comparative genomics and phylogenetic analysis were performed. The *L. rhinocerotis* genome is widely composed of sesquiterpenoid biosynthesis genes. Moreover, the genome of *L. rhinocerotis* appears to code for 1,3-β- and 1,6-β- glucans, as well as lectin, laccase, and other fungal immune-modulatory proteins (FIPs).

Subsequently, proteomic profiling of *L. rhinocerotis* sclerotial proteins were carried out using various spectrometers. Table 1 shows the genomic and proteomic studies of *L. rhinocerotis*. While some of the proteins were unknown, a majority of the proteins were detected as lectin (39.13%) which were speculated for defense mechanisms in *L. rhinocerotis*. The other proteins identified from the study were of pharmacological interest, for example, FIPs (2.52%), antioxidant proteins i.e., manganese superoxide dismutase (Mn-SOD, 0.91%) and glutathione S-transferase (GST, 0.54%). There is a paucity of studies of the myconutrients in this mushroom. Furthermore, it is also necessary to study the molecular and genetic basis of the identified components, and the medicinal and/or nutraceutical properties of *L. rhinocerotis*.

**Table 1:** The genomic and proteomic studies of *L. rhinocerotis*.

**Study**	**Method**	**Findings**	**References**
Genomics	Sesquiterpenoid biosynthesis	Genome coded for 1,3-β- and 1,6-β- glucans, lectin, laccase, and other fungal immune-modulatory proteins (FIPs).	Yap et al., 2014
Proteomics	Two dimensional gel electrophoresis (2DE) coupled with matrix-assisted laser desorption/ionization mass spectrometry (MALDI-MS) and liquid chromatography-mass spectrometry (LC-MS)	Proteins detected were lectin (39.13%), FIPs (2.52%) and antioxidant proteins (1.45%)	Yap et al., 2015

### Nutritional composition of *L. rhinocerotis*

Analysis of the chemical compositions of *L. rhinocerotis* was carried out by Yap et al. (2013). The wild strain (WT Rhino) and the commercial strain (TM02) sclerotial powder were analyzed based on the Association of Official Analytical Chemists (AOAC) procedures. In general, the nutrient composition of the sclerotium of cultivated strain was higher when compared to the wild strain.

The major constituents of *L. rhinocerotis* sclerotia were carbohydrates (monosaccharides and disaccharides) while the fat content was <1%. As reported by Lau et al. (2013a), the beta (β)-glucans represented the dominant glucans in the aqueous extracts of *L. rhinocerotis*, which was 82–93% of total glucan (w/w). The protein content of the sclerotium of TM02 was 3.6-times higher than that of the wild-type. The essential amino acid content (g/kg dry weight) of the commercial strain was 4-times higher than that in the wild strain. The minerals (calcium, potassium, sodium, and magnesium) were also higher in the cultivated strain. Table 2 shows the chemical compositions of *L. rhinocerotis*.

**Table 2:** The chemical and nutritional compositions of *L. rhinocerotis* (Lau et al., 2013a; Yap et al., 2013).

**Composition**	***Lignosus rhinocerotis***	
	**Wild strain (WT Rhino)**	**Commercial strain (TM02)**
Energy (kcal/kg dry weight)	21.80 ± 1.20	32.19 ± 0.71
**NUTRITIONAL COMPOSITION (G/KG DRY WEIGHT)**		
Carbohydrate	8.84 ± 0.35	7.76 ± 0.04
Total sugar	0.07 ± 0.04	0.30 ± 0.10
Protein	0.38 ± 0.03	1.38 ± 0.02
Fat	0.03 ± 0.01	0.08 ± 0.00
**MINERAL (MG/KG DRY WEIGHT)**		
Calcium, Ca	0.37 ± 0.17	1.93 ± 0.60
Potassium, K	13.22 ± 0.56	20.32 ± 2.53
Sodium, Na	0.85 ± 0.07	0.88 ± 0.09
Magnesium, Mg	7.58 ± 0.37	14.79 ± 0.31
**Total glucans (mg/g extract)**	***Lignosus rhinocerotis*** **(KUM61075)**	
	**Hot water extract**	**Cold water extract**
α-glucan	10.37 ± 0.21	2.507 ± 0.30
β-glucans	38.93 ± 9.65	34.87 ± 8.18

### Therapeutic values of *L. rhinocerotis* anti-asthmatic activity

The efficacy of *L. rhinocerotis* in treating asthmatic symptoms has been validated in several *in vitro* and *in vivo* studies. The effects of *L. rhinocerotis* extracts on ovalbumin-induced allergic asthma in *Sprague-Dawley* rats were investigated (Malagobadan et al., 2014). The treatment with *L. rhinocerotis* extracts significantly reduced asthmatic parameters, for instance, the total immunoglobulin E (IgE) in serum, and T-helper type 2 (Th2) cytokine levels (IL-4, IL-5, and IL-13) in bronchoalveolar lavage fluid (BALF). It also inhibited the number of eosinophil in BAFL and diminished the infiltration of eosinophil in the lungs.

A study on airway inflammation in an asthmatic model was reported (Johnathan et al., 2016). The hot aqueous extract of *L. rhinoceroti*s (500 mg/kg) was effective in reducing asthma-related parameters. The extract significantly ameliorated the increase of the total IgE (14.9 ng/ml) in serum, as well as IL-4 (16.2 pg/ml), IL-5 (38.8 ng/ml), and IL-13 (80.5 pg/ml) levels in BALF. It also effectively decreased eosinophils numbers in BALF while attenuating eosinophil infiltrations in the lungs. Sequential extraction using five solvents, i.e., petroleum ether, diethyl ether, hexane, ethyl acetate, and methanol, was conducted prior to gas chromatography-mass spectrometry (GC-MS) analysis (Johnathan et al., 2016). GC-MS examination uncovered five major groups (alkane, fatty acids, benzene, phenol, and dicarboxylic acid) with a total of 18 constituents in *L. rhinoceroti*s. Linoleic acid, octadecane, and 2,3-dihydroxypropyl elaidate were present in abundance in the *L. rhinoceroti*s extract (Johnathan et al., 2016).

### Anti-coagulant and fibrinolytic activities

Cardiovascular diseases can be caused by thrombosis due to fibrin aggregation in the blood (Lee et al., 2005). Natural anti-coagulant and fibrinolytic agents have been used to treat thrombolytic conditions, especially edible mushrooms. Recently, the edible mushroom “wood ear fungus,” *Auricularia polytricha* (Mont.) Sacc was found to be able to produce protease-like fibrinolytic enzymes (Mohamed Ali et al., 2014).

The anti-coagulant activity of the crude aqueous extract of *L. rhinocerotis* was also reported. Kho (2014) demonstrated that the crude extracts of *L. rhinocerotis* caused a 1.2 cm-lytic zone in a fibrin plate assay. Subsequently, aqueous two phase system (ATPS) was employed to partition, purify, and concentrate the protein fraction of the mushroom extract. As a result, a fibrinolytic enzyme with a specific activity of 151.61 U/mg was isolated and the molecular size was estimated to be between 55 kDa and 60 kDa. The anti-platelet activity of the aqueous extract of *L. rhinocerotis* was also reported using fresh human blood (Teo, 2014). The crude extract, after subjected to ATPS, yielded a partially purified protease-like enzyme with size ranging from 50 to 55 kDa.

Sidek Ahmad et al. (2014) tested six different wild sclerotia (LR1 to LR6) of *L. rhinocerotis* obtained from Perak, Malaysia, for their fibrinolytic activities. Out of the six sclerotia used, the LR1 sclerotium exhibited proteolytic activity with a clear zone of 1.31 cm diameter in skim milk agar plates while giving a clear zone of 0.97 cm when tested with fibrin plates. It is presumed that the fibrinolytic activity of *L. rhinocerotis* is attributed to the presence of a protease.

### Anti-inflammatory activity

The anti-inflammatory activity of the sclerotium of *L. rhinocerotis* was previously reported with its hot aqueous, cold aqueous, and methanol extracts (Lee et al., 2012b, 2013b). Lee et al. (2014) reported that the three extracts of *L. rhinocerotis* exhibited anti-inflammatory properties as shown by the carrageenan-induced paw edema test using *Sprague-Dawley* rats. The cold aqueous extract, the most potent extract, was subjected to separation by Sephadex G50 gel filtration chromatography. The resulting high-molecular-weight protein fraction was further assessed for anti-inflammatory activity in lipopolysaccharide (LPS)-induced RAW 264.7 macrophage cells. The protein fraction was shown to inhibit tumor necrosis factor alpha (TNF-α) production.

The anti-inflammatory effect of *L. rhinocerotis* hot aqueous and ethanol extracts on RAW 264.7 cells was further tested (Baskaran et al., 2012; Baskaran, 2015). The ethanol extract showed significant decrease (48.3–88.5%) of nitric oxide (NO) production from 0.01 to 100 μg/mL dose-dependently but the aqueous extract did not show a significant reduction. The ethanol extract was able to activate signal transducer and activator of transcription 3 (STAT3) pathway by reducing inducible nitric oxide synthase (iNOS) and cyclooxygenase-2 (COX-2) expressions while increasing the interleukin 10 (IL-10) expression.

Nallathamby et al. (2016) analyzed the ethyl acetate fraction from the ethanol extract of *L. rhinocerotis*. The fraction significantly reduced the NO production in microglial (BV2) cells by 12 to 70% at 10 and 100 μg/mL; respectively. The major compounds of the ethyl acetate fraction were revealed as linoleic acid, oleic acid, and ethyl linoleate. The identified compounds were further tested individually for their anti-inflammatory activities. Treatment with linoleic acid significantly suppressed iNOS and COX2 expression by 1.2-fold as compared to the control. In another study, LPS-induced BV2 cells pretreated with hot aqueous extract (500 μg/mL), n-butanol fraction of hot aqueous extract (250 μg/mL), and ethyl acetate fraction of hot aqueous extract (250 μg/mL), showed maximum inhibition of NO production by 88.95, 86.50, and 85.93%, respectively (Seow et al., 2017). These studies represent the first evidence of anti-inflammatory properties of *L. rhinocerotis* using brain microglial BV2 cells.

### Anti-microbial activity

Four extracts of the wild *L. rhinocerotis* sclerotium, i.e., petroleum ether, chloroform, methanol, and aqueous extracts, were screened for their anti-microbial properties (Mohanarji et al., 2012). The four extracts were tested against 15 pathogenic bacteria, including *Staphylococcus, Corynebacterium, Bacillus, Streptococcus, Klebsiella, Salmonella, Pseudomonas, Escherichia*, and *Micrococcus* spp.; as well as four fungi species including *Candida* spp and *Mucor* sp. Antifungal and antibacterial activities of the extracts were evaluated by measuring the inhibition zone using disc diffusion assay. The methanol and aqueous extracts (30 mg/mL) showed significant inhibition against the tested microbes except for *Streptococcus pyogenes* and *Serratia marcescens*. A qualitative phytochemical analysis showed the presence of alkaloids, protein, gums and mucilage, and flavonoids in the extracts of *L. rhinocerotis* (Mohanarji et al., 2012).

### Anti-obesity and hepatoprotective activities

A study by Hoe (2014) provided evidence that the aqueous extract of *L. rhinocerotis* mitigated non-alcoholic fatty liver disease in high-fat-diet induced obese hamsters. Further, the extract inhibited weight gain in high-fat-diet fed hamsters. The *L. rhinocerotis* extract did not show any side effects in the experimented animals with regards to the weight of organs i.e., brain, heart, thymus, liver, kidney, adrenal glands, spleen, testicles, and epididymis.

The *L. rhinocerotis* at low dose demonstrated the highest effect in reducing the weights of liver and adipose tissues with no histological abnormalities found in the organs and tissues. The serum biochemical, as well as the liver and kidney function parameters, were also measured. The results showed that the extract reversed the serum levels of several biochemical parameters in the hamsters. The *L. rhinocerotis* aqueous extract was also shown to inhibit reactive oxygen species (ROS) production and reduced cluster of differentiation 68 (CD68) and COX2 expressions in the liver. The extract also downregulated mRNA expressions of different genes, namely TNF-α, IL-1β, IL-6, transforming growth factor beta (TGF-β), and collagen-type 1 (Colla1)-related inflammation and collagen-type 1 (Colla1)-related inflammation and fibrosis caused by obesity.

### Antioxidant properties

The cold aqueous, hot aqueous, and methanol extracts of wild and cultivated sclerotium of *L. rhinocerotis* were assessed for their antioxidant capacities (Yap et al., 2013). The total phenolic content (TPC) of the extracts were calculated in terms of gallic acid equivalents (mg GAE/g). The TPC of the extracts ranged from 19.3 to 29.4 mg GAE/g extract. The ability of the extracts to reduce ferric ions and scavenge free radicals was also measured by using Ferric reducing antioxidant power (FRAP) assay and DPPH• (1,1-diphenyl-2-picrylhydrazyl) radical scavenging assay; respectively. The FRAP value was between 0.006 to 0.016 mmol/min g extract. The DPPH radical scavenging values were 0.05–0.2 mmol Trolox equivalents (TE)/g. The extracts from wild sclerotium of *L. rhinocerotis* had higher antioxidant capacity compared to the cultivated sclerotium. The methanol extract displayed higher activity compared to the aqueous extracts. It is noteworthy to mention that both strains exhibited significantly higher superoxide anion radical scavenging activity when compared to rutin.

Similar analyses were done using aqueous extract of *L. rhinocerotis* (Hoe, 2014). The extract showed half maximal effective concentration (EC_50_) values of DPPH scavenging and ferrous ion chelating activity at 135.16 ± 2.47 mg/mL and 503.34 ± 10.44 mg/mL; respectively. The TPC and flavonoid contents were 366.23 ± 5.06 mg GAE and 28.67 ± 2.5 mg rutin equivalent/g; respectively. In a study by Suziana Zaila (2013), the ferric reducing capacity of pressurized methanol extract was higher than the aqueous extract.

The free radical scavenging abilities, reducing properties, metal chelating activities, and inhibitory effects of lipid peroxidation by *L. rhinocerotis* extracts of mycelium from submerged cultivated and the culture broth were reported by Lau et al. (2014). The aqueous methanol extracts from the mycelium and culture broths showed comparable antioxidant effects with the aqueous methanol extracts of sclerotium. The results suggested that the mycelia of *L. rhinocerotis* obtained in submerged cultivation can also be a valuable source of antioxidant compounds.

The antioxidant activity of hexane and ethyl acetate fractions obtained from ethanol sclerotial extracts of *L. rhinocerotis* were determined (Nallathamby et al., 2014, 2016). The ethanol extract at 5 mg/mL had the highest antioxidant activity among the three extracts with ferric reducing ability of 122.6 mmol ferrous sulfate equivalent (FSE)/g extract. The 2,2′-azino-bis (3-ethylbenzothiazoline-6-sulphonic acid) (ABTS•^+^) and DPPH radical scavenging activity were 86.5 ± 4 mg TE /g and 29.4 ± 1.7%; respectively. The hexane and ethyl acetate fractions had moderate antioxidant activities compared to methanol /ethanol extract.

### Anti-tumor/anti-cancer activities

The traditional claims that *L. rhinocerotis* had anti-tumor/anti-cancer properties were investigated in several cancer and solid tumor cell lines. The first study on the anti-tumor properties of *L. rhinocerotis* sclerotium specified that the water-soluble polysaccharide–protein complex (PR-HW) and alkali-soluble β-glucan (PR-CA) possessed notable host-mediated anti-tumor activity on the Sarcoma 180 implanted male BALB/c mice (Lai, 2005). The PR-HW exhibited apoptotic effect on human acute promyelocytic leukemia cells (HL-60), human chronic myelogenous leukemia cells (K562), and human acute monocytic leukemia cells (THP-1) with IC_50_ of 100 mg/ml, 400 mg/ml, and >400 mg/ml; respectively after 72 h of incubation (Lai, 2005; Lai et al., 2008). Besides, flow cytometric analysis revealed that the onset of apoptosis could be linked to the cell cycle arrest at Gap1 (G1) phase.

The cold-water extract (LR-CW) prepared from the sclerotium of *L. rhinocerotis* exhibited antiproliferative activity in human breast carcinoma cells (MCF-7) and human lung carcinoma cells (A549), with IC_50_ of 96.7 and 466.7 μg/mL, respectively. In contrast, the LR-CW did not show significant cytotoxicity toward the two corresponding normal human cells, i.e., human breast cells (184B5) and human lung cells (NL 20). From the results of DNA fragmentation assay, the cell death was attributed to apoptosis. The high-molecular-weight fraction of the cold water extract exhibited cytotoxicity toward MCF7 and A549 cancer cells, with IC_50_ of 70.0 and 76.7 μg/mL, respectively (Lee et al., 2012a).

Subsequently, the cytotoxicity of hot aqueous and cold aqueous extract of *L. rhinocerotis* sclerotium were screened using 11 human cell lines, namely HL-60 (human acute promyelocytic leukemia cells), MCF7, MDA-MB-231 (human breast adenocarcinoma cells), HCT116 (human colorectal carcinoma cells), PC-3 (human prostate adenocarcinoma cells), A549 (human lung carcinoma cells), MRC-5 (human lung fibroblast cells), HepG2 (human hepatocellular carcinoma cells), WRL68 (human embryonic liver cells), HSC2 (human squamous carcinoma cells), and HK1 (human nasopharyngeal carcinoma cells) (Lau et al., 2013a). The cold aqueous extract was cytotoxic toward solid tumor cells with IC_50_ of 37–120 mg/mL, whereas the hot aqueous extract was inactive toward the solid tumor cells.

The pressurized methanol extract exerted a higher cytotoxic activity (IC_50_: 600 μg/mL) when compared to the pressurized aqueous extract (IC_50_: 1200 μg/mL) in HCT 116 (human colorectal carcinoma) cells as determined by 3-(4,5-Dimethylthiazol-2-yl)-2,5-diphenyltetrazolium bromide (MTT) assay (Suziana Zaila, 2013). Both extracts did not exert cytotoxic activity on CCD-18co (human normal colon fibroblast cells) and V79-4 (Chinese hamster lung fibroblast cells) (IC_50_> 2000 μg/mL). The mode of cell death induced by methanol and aqueous extracts was primarily apoptosis as assessed by Annexin V-fluorescein isothiocyanate/propidium iodide (FITC/PI) dual staining. Both extracts arrested HCT 116 cells at G0/G1 phases with the corresponding decrease of S-phase population.

Lai et al. (2014) indicated that the polysaccharide at concentrations ranging from 4 to 8 μg/mL of *L. rhinocerotis* (collected from the forest in Kuala Lipis, Pahang, Malaysia) inhibited 45% growth of human lung carcinoma cells (A549). This is 100-fold lower than the results reported by Lee et al. (2012a). A preliminary study found that the antiproliferative ingredient in the polysaccharide extract was the β-glucan.

The mycelial extract of *L. rhinocerotis* cultured in a stirred tank reactor was also reported to exhibit cytotoxic effect on cervical cancer cells (Ca Ski) (Abdullah et al., 2010). The ammonium sulfate precipitate obtained from the mycelial protein fraction showed a growth inhibition of 72–82% against Ca Ski cells. The aqueous methanol extract of mycelial and culture broth of *L. rhinocerotis* from liquid fermentation did not exert cytotoxicity toward the normal cell lines (Lau et al., 2014).

In conclusion, there is a consistency among the reported studies, that the alcoholic extracts were not cytotoxic toward various cancer cell lines (Yap et al., 2013; Lau et al., 2014). However, the selectivity of the extracts on non-tumorigenic cells lines is not consistent (Lau et al., 2013a; Lee et al., 2014). The cultivation, processing, and preparation of extract may affect the cytotoxicity toward different cell lines. The chemical compositions of the extract may also influence the degree of cytotoxic level toward the cell lines. According to the reported literature, the primary identified bioactive components are more to high-molecular-weight hydrophilic components (Lai et al., 2008; Lau et al., 2014), protein-carbohydrates complex (Lee et al., 2014), and protein/peptides (Lau et al., 2013b).

### Anti-viral activity

The mycelial protein fractions of *L. rhinocerotis* were previously reported to inhibit human papilloma virus (HPV) activity (Abdullah et al., 2010). The anti-dengue activity tested by plaque reduction assay against dengue virus type-2 (DENV-2) strain was detected in hot aqueous extract of *L. rhinocerotis* at IC_50_ of 520 μg/mL (Ellan et al., 2013). Hot aqueous extract of *L. rhinocerotis* also inhibited dengue viral RNA synthesis by 99.68%. The extract showed anti-dengue activity in viral penetration assay but did not have a significant effect on DENV-2 virucidal activity and viral attachment. The anti-dengue activity was correlated with the carbohydrate content in the hot aqueous extract of *L. rhinocerotis* (2.0 mg/mL) (Ellan et al., 2014). This suggested that the *L. rhinocerotis* has anti-dengue activity attributed to the polysaccharide content in the sclerotium.

### Immunomodulatory actions

Immune modulatory activity can lead to anti-inflammation and anti-tumor effects. The immune responses are mediated by various immune cells and their secondary secretory components (Wong and Cheung, 2009). Guo et al. (2011) investigated the effect of aqueous extract of *L. rhinocerotis* sclerotium on RAW 264.7 and primary macrophages isolated from BALB/c mice. The mushroom treatment promoted a significant up-regulation of pinocytosis, leading to an increase in the production of ROS, NO, and TNF-α production. The iNOS expression in both RAW 264.7 cells and primary macrophages was also increased. The expression of Dectin-1^+^ cells on the cell surface was shown to decrease. On the other hand, the complement receptor (CR3^+^) and toll-like receptor (TLR2^+^) were increased in response to aqueous extract-treated primary macrophage. The aqueous extract also increased the phosphorylation of I-kappa-B alpha (IKBα), which then triggered the nuclear factor kappa B (NF-κB) signaling pathway. Therefore, the immunomodulatory effect of *L. rhinocerotis* could be intervened by macrophage activation *via* the NF-κB signal pathway.

The *in vitro* results from the previous studies were further tested in *in vivo* studies using healthy BALB/c mice and athymic nude mice. Their serum cytokine profile, splenocytes, and peritoneal exudate cells (PECs) were analyzed (Wong et al., 2011). The hot water extract and sonication-assisted cold alkali-soluble polysaccharides (PRS) of *L. rhinocerotis* increased the spleen weight of both healthy BALB/c mice and athymic nude mice. The hot water extract activated the neutrophil production whereas the PRS stimulated other innate immune cells.

The combined effect of *L. rhinocerotis* supplementation and resistance training on immune functions/parameters such as CD3^+^ and CD4^+^T lymphocytes; and CD4^+^ B lymphocytes were measured in young males between 19 to 25 years (Chen et al., 2016). The results indicated that the combination of extracts and resistance training did not affect the immune functions of the subjects.

In another study, a water-soluble polysaccharide-protein complex (PRW1) was isolated from the sclerotium of *Polyporus rhinocerus* Cooke (synonym of *L. rhinocerotis*) and further purified by membrane ultrafiltration (Liu et al., 2016). It showed a significant increase of NO production and enhanced the release of a variety of cytokines, for example, granulocyte colony stimulating factor (GCSF) and granulocyte-macrophage colony-stimulating factor (GMCSF). Accompanying the release of stimulating factors were the IL-6, IL12p40/70, monocyte chemoattractant protein-1 (MCP-1), MCP-5, macrophage inflammatory protein (MIP-1-α), MIP-2, regulated on activation normal T cell expressed and secreted (RANTES), soluble tumor necrosis factor receptor I (sTNFRI), and TNF-α. *L. rhinocerotis* also triggered extracellular signal-regulated kinase (ERK) phosphorylation and increased the iNOS expression. It was the first report that provided the molecular mechanism of the immunostimulatory properties of *Polyporus*-derived protein complex on RAW 264.7cells. *Polyporus rhinocerus* sclerotium may have a potential application for cancer immunotherapy.

It is noteworthy to clarify that while the myconutrients of *L. rhinocerotis* can improve immune system by activating several immune effector cells (pro-inflammatory), they too, can suppress the immune system by inhibiting certain inflammation markers (anti-inflammation). This “immunomodulating properties” is very common among fungal metabolites, especially the mushroom polysaccharides.

### Neuritogenic properties

The sclerotium of *L. rhinocerotis* was investigated for neurite outgrowth in rat pheochromocytoma adherent (PC12 adh) cells (Eik et al., 2011). The aqueous extract increased the percentage of neurite bearing cells by 9.8 to 23.6% in PC12 cells. The aqueous and ethanol extracts stimulated maximal neurite outgrowth of 23.6% and 18.5%; respectively at 20 μg/mL. It is also shown that the *L. rhinocerotis* aqueous extract showed better neurite outgrowth compared to other medicinal mushrooms tested. Furthermore, the combination of 20 μg/mL aqueous extract and 30 ng/mL of nerve growth factor (NGF) enhanced the neurite outgrowth by 42.1% compared to either aqueous extract (24.4%) or NGF (24.6%) alone (Eik et al., 2012).

The aqueous extract of the *L. rhinocerotis* mycelium also had high neurite outgrowth activity of 21.1% at 20 μg/mL in PC12 cells (John et al., 2011). The synergistic effects of *L. rhinocerotis* mycelium extract combined with other natural products were further tested on PC12 cells. The treatments with the combination of aqueous extract (20 μg/mL) and *Gingko biloba* (30 μg/mL) resulted in 39.89% of neurite outgrowth activity when compared to *G. biloba* alone (31.15%) (John et al., 2012). The increase of neurite outgrowth activity was significant although the synergistic effects are not remarkably high. The effects of *L. rhinocerotis* mycelium extract (20 μg/mL) bio-augmented with 1 μg/mL of curcumin were subsequently tested (John et al., 2013). The combination has enhanced neurite outgrowth by 27.2% when compared to curcumin alone.

Phan et al. (2013) reported neurite outgrowth of aqueous extract of *L. rhinocerotis* sclerotium and mycelium in mouse neuroblastoma (N2a) cells. At 20 μg/mL, the aqueous extract of sclerotium resulted in 38.1% of neurite bearing cells, which was approximately twice the number of NGF-treated neurite bearing cells. However, the aqueous extract of the mycelium did not cause a significant increase in neurite outgrowth when compared to NGF treatment. In all the studies, neuronal differentiation in the cell lines treated by *L. rhinocerotis* extracts were further demonstrated by indirect immunofluorescence staining of neurofilament protein.

According to the studies by Seow et al. (2013a, 2015), the maximal neuritogenic activity in PC12 at 25 μg/mL of aqueous extract was 20.99% followed by ethanolic extract (17.4%) and crude polysaccharides (16.4%). The hot aqueous extract (25 μg/mL) stimulated neuritogenesis as equivalent to NGF (50 μg/mL). However, all the extracts promoted neuritogenesis without stimulating the release of NGF in PC12 cells. The tyrosine kinase (Trk) and ERK1/2 inhibitors (K252a, U0126, and PD98059) showed a decrease in the neurite bearing cells percentage by 82.2, 86.2, and 91.6% in NGF-treated cells; and 80.9, 86.7, and 84.6% in hot aqueous extract-treated cells. The NGF-mimicking potential of *L. rhinocerotis* is postulated to follow the phosphoinositide 3-kinase protein kinase B (P13K-Akt) and ERK1/2 pathways (Eik et al., 2012; Seow et al., 2013b).

On the other hand, the ethyl acetate and n-butanol fractions of *L. rhinocerotis* were found to mimic the neuritogenic activity of NGF by targeting the TrkA receptor and activated the mammalian target of rapamycin (mTOR) signaling pathway with the phosphorylation of the transcription factors, cyclic adenosine monophosphate (cAMP) response element-binding protein (CREB), and leading to the increased expressions of neuritogenesis biomarker, i.e., the growth associated protein 43 (GAP43), tubulin alpha 4A (TUBA4A), and tubulin beta 1 (TUBB1) in PC12 cells (Seow, 2016).

In a different study, aqueous extract of *L. rhinocerotis* sclerotium was investigated for stimulation of neurite outgrowth in dissociated cells of the brain, spinal cord, and retina of chick embryo (Samberkar et al., 2015). After 48 h incubation, the aqueous extract at 50 μg/mL induced maximum neurite outgrowth of 20.8 and 24.7% in brain and spinal cord. On the other hand, 20.8% of neurite outgrowth was achieved in retinal cells at 25 μg/mL. The neuronal differentiation by *L. rhinocerotis* aqueous extract-treated cells was then confirmed by immunofluorescence staining.

### Toxicology evaluation

It is crucial that the toxicity assessment is done both in *in vitro* and *in vivo* assays in order to develop *L. rhinocerotis* as health supplements. The teratogenicity effects of *L. rhinocerotis* was carried out by Lee et al. (2013a). The freeze-dried sclerotium of *L. rhinocerotis* was orally administrated to both male and female mice for 28 days and then the animals were allowed to mate for 10 days. The female rats were continuously fed with the sclerotium powder until the pups were delivered (around 7–8 weeks after mating period). The pups were assessed for anti-fertility and teratogenic effects, if any. The 100 mg/kg sclerotium of *L. rhinocerotis* did not affect the fertility of the rats. Further, the dosage tested did not trigger teratogenic effects or aberrations in the litter.

The genotoxic effects of the sclerotium of the *L. rhinocerotis* or its possibility to cause gene mutations were evaluated in the plate incorporation and pre-incubation tests (Lee et al., 2013a). The bacteria strains were *Salmonella typhimurium* and *Escherichia coli* strains. There was no genotoxic effects or mutation in all the five strains (TA98, TA100, TA1535, TA1537, and WP2 uvrA) tested.

The genotoxic effects of *L. rhinocerotis* sclerotia were further evaluated by the bacterial reverse mutation or Ames test, the *in-vitro* chromosome aberration and *in vivo* mammalian erythrocyte micronucleus assays (Chen et al., 2013). At the concentration of the mycelium of *L. rhinocerotis* at 100 mg/mL (5 mg/plate) dose no mutagenic activity in the presence and absence of S9 metabolic activation system was observed in all the five *Salmonella* strains. The number of structural aberrations in Chinese hamster ovary (CHO-K1) cells was comparable to the negative control. The cells were unaltered and frequencies across the treatment had insignificant damage. The treatment with *L. rhinocerotis* displayed no adverse effect on the natural micronucleus frequency in both male and female mice. Further polychromatic erythrocytes (PCE) percentage and micronucleus frequency in 2,000 mg/kg *L. rhinocerotis* orally administrated animals had no significant difference when compared to the control animals.

The effect on cellular viability of *L. rhinocerotis* extracts of non-tumorigenic cell lines was investigated. The aqueous extract of *L. rhinocerotis* sclerotium was not cytotoxic to normal human breast (184B5) and normal lung (NL20) cells (Lee et al., 2012a). The methanol extract of *L. rhinocerotis* sclerotium also was not cytotoxic to normal human colon (CCD-18co), kidney (HEK-293), nasopharyngeal (NP69), oral (OKF6), rat kidney (NRK-52E), and Vero cell lines (Lau et al., 2013a; Suziana Zaila, 2013).

Eik et al. (2012) and Seow et al. (2015) reported that the aqueous and ethanol extracts of the *L. rhinocerotis* sclerotium were non-cytotoxic to PC12 cells after 48 h incubation. Phan et al. (2013) also reported that the aqueous extracts of the mycelium and sclerotium of *L. rhinocerotis* were not cytotoxic with IC_50_ values of 1.75–5.93 mg/mL to mouse embryonic fibroblasts (BALB/3T3) and N2a cells after 24 h incubation. Baskaran (2015) reported that the aqueous and ethanol extracts were non-cytotoxic to RAW264.7 macrophages cells. Nallathamby et al. (2013, 2016), too found that the ethanol, hexane, and ethyl acetate fractions obtained from the sclerotium was non-cytotoxic to BV2 microglial cells. The crude polysaccharides, hot aqueous and ethanol extracts of the sclerotium of *L. rhinocerotis* were shown to be non-cytotoxic to BV2 microglial cells. Further, the hexane, ethyl acetate, n-butanol and aqueous fractions from hot aqueous and ethanol extracts were not cytotoxic to BV2 microglial cells (Nallathamby et al., 2013, 2016).

In a sub-acute toxicity *in vivo* study using Sprague Dawley rats, the sclerotium powder of cultivated and wild *L. rhinocerotis* were orally administrated at 250, 500, and 1,000 mg/kg doses (Shien et al., 2011). Neither the cultivated nor the wild *L. rhinocerotis* sclerotium had adversative effects on the growth rate, blood, and clinical biochemical parameters. There were also no pathological changes in the vital organs like liver, kidneys, spleen, and lungs.

Findings obtained from the chronic toxicity study on *L. rhinocerotis* TM02 cultivar were also similar (Lee et al., 2013a). The oral administration of the sclerotium powder at the highest dose of 1,000 mg/kg did not cause adverse effects. Therefore, the no-observed-adverse-effect-level (NOAEL) dose of the sclerotium powder was higher than 1,000 mg/kg. Based on both *in vitro* and *in vivo* toxicity assessments reported, indicate that cultivated *L. rhinocerotis*, the mycelium or sclerotium, were safe for consumption.

### Future perspectives

Table 3 summarizes the medicinal properties of this mushroom. In order to expand the application of this mushroom, certain aspects of research should be further studied in detail.

**Table 3:** The medicinal properties of *L. rhinocerotis* (a summary).

**Therapeutic value**	**Origin**	**Mushroom part**	**Extracts**	**Bioactive components**	**Experimental models**	**Assay**	**Findings**	**References**
Anti-asthmatic activity	–	–	–	–	*In vivo*	Albumin induced allergic asthma study	Reduced IgE in serum; Th2 cytokines, suppressed BALF	Malagobadan et al., 2014
	Cultivated	Sclerotium	Hot aqueous	Alkane, fatty acids	*In vivo*	Albumin induced allergic asthma study	Ameliorated the increase in total IgE, IL-4, IL-5, and IL-13 levels in BALF; suppressed eosinophils numbers in BALF	Johnathan et al., 2016
Anti-coagulant and fibrinolytic activity	Wild	Sclerotium	Aqueous	Protease	*In vitro*	Fibrin plate method	Moderated fibrinolytic activity	Sidek Ahmad et al., 2014
	Cultivated	Sclerotium	Tris-HCl buffer	Proteins	*In vitro*	Anticoagulant assay, Anti-platelet assay	Exhibited anti-coagulant and anti- platelet activity at extract concentration of 5–25 mg/mL	Teo, 2014
	Cultivated	Sclerotium	Tris-HCl buffer	Proteins	*In vitro*	Fibrin plate method, Folin spectroscopy method	Produced lytic zone at extract concentration of 0.3–0.7 mg/mL	Kho, 2014
Anti-inflammatory properties	Cultivated	Sclerotium	Hot aqueous, Cold aqueous methanol	Polysaccharide	*In vitro In vivo*	TNF-α production (ELISA kit), Carrageenan-induced paw edema and cotton pellet induced granuloma studies	Inhibited TNF-α production in LPS-induced RAW264.7; reduced carrageenan induced paw edema in rats	Lee et al., 2014
	Cultivated	Sclerotium	Hot aqueous, ethanol	-	*In vitro*	NO assay, cytokine assay	Reduced NO in LPS-stimulated BV2 cells; reduced iNOS and COX2 at 10 ug/mL	Nallathamby et al., 2016
	Cultivated	Sclerotium	Ethanol, hexane, ethyl acetate	Fatty acids	*In vitro*	NO assay, cytokine determination (qPCR)	Reduced NO production in RAW 264.7 cells; reduced cytokine effects and activated STAT3 pathway	Baskaran, 2015
Anti-microbial activity	Wild	Sclerotium	Petroleum ether, chloroform, methanol	–	*In vitro*	Disc diffusion assay	Displayed antifungal and antibacterial activities	Mohanarji et al., 2012
Anti-obesity activity	Cultivated	Sclerotium	Hot aqueous	–	*In vivo*	Serum biomarkers, ROS, Western blot, qPCR expressions	Prevented fatty liver disease in HFD induced obesity; reduced obesity and body fat; improved serum parameters and liver lipid metabolism.	Hoe, 2014
Antioxidant properties	Cultivated and wild	Sclerotium	Hot aqueous, Cold aqueous methanol	N.D.	*In vitro*	DPPH, ABTS, SOA radical scavenging assay, FRAP, Folin-Ciocalteu, xanthine oxidase assay	Exhibited radical scavenging and reducing capacity; the wild strain showed higher antioxidant activity	Yap et al., 2013
	Wild	Sclerotium	Aqueous, Methanol	N.D.	*In vitro*	FRAP assay	Exhibited reducing capacity	Suziana Zaila, 2013
	Cultivated	Mycelium, Culture broth, Sclerotium	Aqueous, Methanol	N.D.	*In vitro*	DPPH, ABTS, FRAP and CUPRAC assays, metal chelating, lipid peroxidation	Mycelium and culture broth showed higher activity than sclerotium	Lau et al., 2014
	Cultivated	Sclerotium	Hot aqueous	N.D.	*In vitro*	DPPH, ferrous irons chelating, Phenolic content and flavonoids	Exhibited scavenging ability; high content of phenolic and flavonoid	Hoe, 2014
	Cultivated	Sclerotium	Ethanol, Hexane, Ethyl acetate	Fatty acids	*In vitro*	DPPH, FRAP, ABTS and TPC	Exhibited antioxidant activity	Nallathamby et al., 2016
Antitumor/ anticancer activity	Cultivated	Sclerotium	Hot aqueous cold alkaline	Polysaccharide protein complex and glucan	*In vitro*	TBE assay, cell cycle analysis	Inhibited growth of human leukemic cells—HL-60, K562, and THP-1	Lai et al., 2008
	Cultivated	Mycelium, Culture broth, Sclerotium	Cold aqueous, Ammonium sulfate percipitate	Protein/ peptide	*In vitro*	NR assay	Cytotoxic against CaSki cells	Abdullah et al., 2010
	Cultivated	Sclerotium	Cold aqueous	HMW fraction protein and protein carbohydrates complexes	*In vitro*	MTT and DNA fragmentation	Cytotoxic against MCF7 and A549 cancer cells; non-cytotoxic to 184B5 and NL 20 nontumorigenic cells	Lee et al., 2012a
	Cultivated	Sclerotium	Hot aqueous cold aqueous	Protein/ peptide	*In vitro*	MTT and BTE assays	Cytotoxic to A549, HepG2, HCT 116, HK1, HSC2, MCF7, MDA-MB-231, PC3, and HL-60 cancer cells and non-tumorigenic MRC5 cells	Lau et al., 2013a
	Cultivated	Sclerotium	Hot aqueous, Cold aqueous methanol	N.D.	*In vitro*	MTT assay	Cold aqueous extracts were cytotoxic against MCF7 cells; hot aqueous and methanol were not cytotoxic	Yap et al., 2013
	Wild and Cultivated	Sclerotium	Aqueous, Methanol	N.D.	*In vitro*	MTT assay	Cytotoxicity against HCT 116 cells; non-cytotoxic against CCD- 18Co cells	Suziana Zaila, 2013
	Cultivated	Mycelium, Culture broth	Aqueous, Methanol	N.D.	*In vitro*	MTT assay	Low or no cytotoxicity against cancer and normal cells	Lau et al., 2014
	Wild	Sclerotium	Crude polysaccharide	beta-glucan-rich polysaccharide	*In vitro*	MTT assay	Hot aqueous and crude polysaccharide extract inhibited growth of A549 cells.	Lai et al., 2014
Anti-viral activity	Cultivated	Sclerotium	Cold aqueous	Proteins	*In vitro*	NR assay	Inhibited HPV activity	Abdullah et al., 2010
	Cultivated	Sclerotium	Hot aqueous	N.D.	*In vitro*	Plaque reduction assay	Exhibited anti-dengue activity	Ellan et al., 2013, 2014
Immunomodulatory actions	Cultivated	Sclerotium	Hot aqueous	Polysaccharide protein complex β- glucan	*In vitro*	MTT, cytokine antibody assay, expression of cell surface β- glucan receptors	Stimulated immune cells, promoted cell proliferation, increased expression of cytokines	Wong and Cheung, 2009
	Cultivated	Sclerotium	Hot aqueous	Polysaccharide	*In vitro*	ROS, NO, TNF-α release, expression of Dectin-1, CR3, TL2, NFκB	Enhanced functional activity of macrophages	Guo et al., 2011
	Cultivated	Sclerotium	Hot aqueous	polysaccharide	*In vivo*	BALB/c mice and BALB/c nude mice	Demonstrated distinctive immunomodulatory effect	Wong et al., 2011
	Cultivated	Sclerotium	Hot aqueous	water-soluble polysaccharide–protein complex	*In vitro*	MTT, NO, cytokine antibody array, Western blot	Significantly induced NO production; enhanced the release of cytokines; triggered ERK phosphorylation to activate macrophages, increased the expression level of inducible NOS.	Liu et al., 2016
Neuritogenic activity	Cultivated	Sclerotium	Hot aqueous	N.D.	*In vitro*	Quantification of neurite bearing cells, Neurofilament staining	Stimulated neurite outgrowth in PC12 cells	Eik et al., 2011, 2012
	Cultivated	Mycelium Culture broth	Hot aqueous	N.D.	*In vitro*	Quantification of neurite bearing cells, Neurofilament staining	Stimulated neurite outgrowth in PC12 cells in combination with curcumin	John et al., 2012, 2013
	Cultivated	Sclerotium Mycelium	Hot aqueous	N.D.	*In vitro*	Quantification of neurite bearing cells Neurofilament staining	Stimulated neurite outgrowth in N2A cells	Phan et al., 2013
	Cultivated	Sclerotium	Hot aqueous, ethanol and crude polysaccharide	N.D.	*In vitro*	Quantification of neurite bearing cells, Neurofilament staining, Protein expression (ELISA), Inhibitor	Stimulated neurite outgrowth in PC12 cells mediated through phosphorylation of Trk A receptor and ERK1/2 pathway	Seow et al., 2015
	Cultivated	Sclerotium	Hot aqueous	N.D.	*In vivo*	Quantification of neurite bearing cells, Neurofilament staining	Stimulated neurite outgrowth in dissociated cells from the brain, spinal cord, and retina from chick embryo	Samberkar et al., 2015

*N.D, not determined; IgE, immunoglobulin E; Th2, T-helper type 2; BALF, bronchoalveolar lavage fluid; IL-4, interleukin 4; IL-5, interleukin 5; IL-13, interleukin 13; Tnf-α, tumor necrosis factor alpha; LPS, lipopolysaccharide; NO, nitric oxide; iNOS, inducible nitric oxide synthase; COX2, cyclooxygenase-2; qPCR, quantitative polymerase chain reaction; STAT3, signal transducer and activator of transcription 3; ROS, reactive oxygen species; HFD, high fat diet; DPPH, 1,1-diphenyl-2-picrylhydrazyl; ABTS, 2,2'-azino-bis (3-ethylbenzothiazoline-6-sulphonic acid); FRAP, ferric reducing antioxidant power; CUPRAC, cupric reducing antioxidant capacity; TPC, total phenolic content; HL-60, human promyelocytic leukemia cells, NR, neural red; HPV, human papilloma virus; CR3, complement receptor 3; TL2, toll-like receptor 2; NFκB, Nuclear factor κB; ERK, extracellular signal-regulated kinase; ELISA, enzyme-linked immunosorbent assay; Trk, tyrosine kinase*.

**Figure 3:**
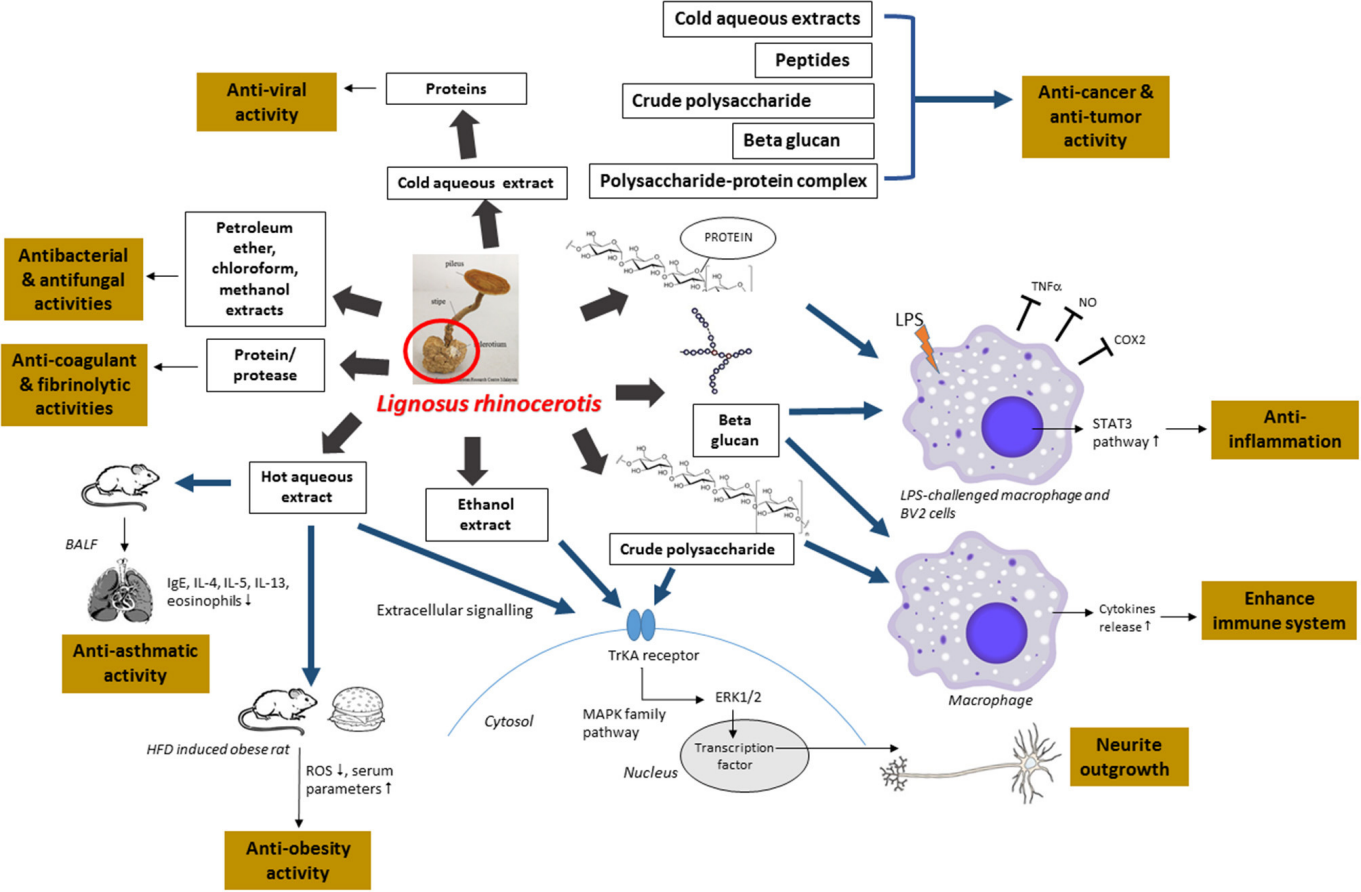
An overview of the recent findings of the medicinal properties of *L. rhinocerotis* and the proposed mechanistic pathways. Ig, immunoglobulin; BALF, bronchoalveolar lavage fluid; IL, interleukin; TNF-α, tumor necrosis factor alpha; LPS, lipopolysaccharide; NO, nitric oxide; COX2, cyclooxygenase-2; STAT3, signal transducer and activator of transcription 3; ROS, reactive oxygen species; HFD, high fat diet; ERK, extracellular signal-regulated kinase; Trk, tyrosine kinase; MAPK, mitogen activated protein kinase.

First, since there is a high demand for this mushroom and the current lack of supply, more studies could be conducted on the domestication of *L. rhinocerotis* by optimizing the cultivation conditions to produce more consistent yield and bioactive components in extracts. Second, in most studies conducted so far, the medicinal properties of *L. rhinocerotis* were demonstrated using crude mushroom extracts or mixture containing different constituents. It is therefore necessary to identify the active bio-molecules for a better grasp of the medicinal properties and the mechanistic pathways of the novel compounds. Figure 3 provides an overview of the recent findings of the medicinal properties of *L. rhinocerotis* and the proposed mechanistic pathways of some of the activities.

Third, many findings were based on *in vitro* results. Thus, these studies should be further investigated in *in vivo* and human/ clinical studies to validate the uses scientifically. Besides, more applications for *L. rhinocerotis* extracts/ bioactive compounds should be ventured such as nanotechnology and genomic identifications. Finally, more surveys should be conducted in cooperation with the indigenous people to discover more traditional medicine applications. This will enhance the understanding of the medicinal properties based on traditional knowledge and applications for evidential documentation.

## Conclusion

The *L. rhinocerotis* sclerotium is well-known for its ethnomedicinal uses in curing many ailments. The efforts to research, compile, and validate the information scientifically is a continuous process in the development of medicinally relevant products. This review showed that the sclerotium of *L. rhinocerotis* possessed several potential therapeutic properties that would be useful to human's health. Further research is warranted to identify and isolate chemical components/bioactive components and their mode of actions.

## Author contributions

NN, C-WP, SL-SS, AB, and VS have written the first draft of the manuscript. HL and SNAM revised and improved the first draft. All authors have seen and agreed on the finally submitted version of the manuscript.

### Conflict of interest statement

The authors declare that the research was conducted in the absence of any commercial or financial relationships that could be construed as a potential conflict of interest.
